# Transcriptome survey of the anhydrobiotic tardigrade *Milnesium tardigradum *in comparison with *Hypsibius dujardini *and *Richtersius coronifer*

**DOI:** 10.1186/1471-2164-11-168

**Published:** 2010-03-12

**Authors:** Brahim Mali, Markus A Grohme, Frank Förster, Thomas Dandekar, Martina Schnölzer, Dirk Reuter, Weronika Wełnicz, Ralph O Schill, Marcus Frohme

**Affiliations:** 1 Molecular Biology and Functional Genomics, University of Applied Sciences Wildau, Bahnhofstraße 1, 15745 Wildau, Germany; 2 Department of Bioinformatics, Biocenter, University of Würzburg, Am Hubland, 97074 Würzburg, Germany; 3 German Cancer Research Center (DKFZ), Protein Analysis Facility, Im Neuenheimer Feld 280, 69120 Heidelberg, Germany; 4 Oncoscience AG, Wedel, Germany; 5 Biological Institute, Zoology, Universität Stuttgart, Pfaffenwaldring 57, 70569 Stuttgart, Germany

## Abstract

**Background:**

The phenomenon of desiccation tolerance, also called anhydrobiosis, involves the ability of an organism to survive the loss of almost all cellular water without sustaining irreversible damage. Although there are several physiological, morphological and ecological studies on tardigrades, only limited DNA sequence information is available. Therefore, we explored the transcriptome in the active and anhydrobiotic state of the tardigrade *Milnesium tardigradum *which has extraordinary tolerance to desiccation and freezing. In this study, we present the first overview of the transcriptome of *M. tardigradum *and its response to desiccation and discuss potential parallels to stress responses in other organisms.

**Results:**

We sequenced a total of 9984 expressed sequence tags (ESTs) from two cDNA libraries from the eutardigrade *M. tardigradum *in its active and inactive, anhydrobiotic (tun) stage. Assembly of these ESTs resulted in 3283 putative unique transcripts, whereof ~50% showed significant sequence similarity to known genes. The resulting unigenes were functionally annotated using the Gene Ontology (GO) vocabulary. A GO term enrichment analysis revealed several GOs that were significantly underrepresented in the inactive stage. Furthermore we compared the putative unigenes of *M. tardigradum *with ESTs from two other eutardigrade species that are available from public sequence databases, namely *Richtersius coronifer *and *Hypsibius dujardini*. The processed sequences of the three tardigrade species revealed similar functional content and the *M. tardigradum *dataset contained additional sequences from tardigrades not present in the other two.

**Conclusions:**

This study describes novel sequence data from the tardigrade *M. tardigradum*, which significantly contributes to the available tardigrade sequence data and will help to establish this extraordinary tardigrade as a model for studying anhydrobiosis. Functional comparison of active and anhydrobiotic tardigrades revealed a differential distribution of Gene Ontology terms associated with chromatin structure and the translation machinery, which are underrepresented in the inactive animals. These findings imply a widespread metabolic response of the animals on dehydration. The collective tardigrade transcriptome data will serve as a reference for further studies and support the identification and characterization of genes involved in the anhydrobiotic response.

## Background

Desiccation tolerance or anhydrobiosis is the ability of an organism to survive almost complete drying without sustaining damage. Anhydrobiosis is observed in certain micro-organisms, plants and animals such as rotifers, brine shrimp cysts, tardigrades and insect larvae, as for example those of the *Polypedilum vanderplanki *[1-3]. Studying the mechanisms of tolerance against desiccation may lead to development of new methods for preserving biological materials, which is of enormous practical importance in industrial as well as in medical fields [4]. In the dry state, the metabolism is suspended and the duration that anhydrobiotic organisms can survive ranges from years to centuries. Tardigrades are able to survive long periods of desiccation [5-8]. The hitherto longest known observation of an extended lifespan of 20 years has been demonstrated in the anhydrobiotic state of the species *Echiniscus testudo *Doyère 1840 [9]. Anhydrobiosis probably depends on a series of complex morphological, physiological and genetic adaptations that involve the stabilization of macromolecular complexes. As a consequence, a number of components have been identified and appear to be important for protecting these organisms from desiccation damage. Among them are the highly hydrophilic LEA proteins, which have been initially described in plants but have been identified in several invertebrates [10,2,12], as well as non-reducing disaccharides like trehalose [13-16]. We are studying anhydrobiosis in the limno-terrestrial tardigrade *Milnesium tardigradum *Doyère 1840 which shows remarkable resistance to adverse environmental conditions in all stages of life [17] - even to extreme levels of ionizing radiation [18] and the vacuum of space in low earth orbit [19]. *M. tardigradum *outperforms several other tardigrade species in tolerance e.g. survival of extreme temperatures above 100°C [20] as well as freezing [21,22]. Similar anhydrobiotic resistance to extreme environmental stress has been observed in other animals such as bdelloid rotifers or chironomid larvae [23,24] suggesting common mechanisms that allow anhydrobiotic survival and conferring radiation tolerance. The tardigrade phylum currently includes more than 1000 species living in the sea, in fresh water and on land. These last, needing at least a film of water to be active, are called limno-terrestrial and include most of the anhydrobiotic species [25]. They have been studied for their fascinating ability to perform anhydrobiosis and consequently serve as a potential model for studying tolerance and survival of multicellular organisms to a variety of extreme environmental conditions. Although there are several physiological, morphological and ecological studies on anhydrobiotic tardigrades [26-30], only limited DNA sequence information from molecular phylogenetic studies is available [30-34]. However, some sequence resources are only available from the species *Hypsibius dujardini *Doyère 1840 [Daub et al. Unpublished data 2003] and *Richtersius coronifer *(Richters 1903) [35]. Studies of *H. dujardini *have been focused mainly on developmental and evolutionary biology [36-38]. In this study we generated 9984 ESTs of *M. tardigradum *from active and inactive (anhydrobiotic/tun) stages, thereby establishing *M. tardigradum *as a model for anhydrobiosis research. These ESTs and the resulting unigenes were functionally annotated using Gene Ontology vocabulary. Furthermore, a cross-species comparison between *M. tardigradum, H. dujardini *and *R. coronifer *has been performed.

## Results and discussion

### cDNA libraries and sequence datasets

We have generated two directionally cloned cDNA libraries from active and inactive stages of *M. tardigradum *and subjected them to single pass Sanger sequencing. Furthermore we retrieved two EST datasets from public sequence databases (see Table 1 and 2). The datasets used in this study consisted of EST sequences from, *M. tardigradum *active and inactive stages, *H. dujardini *[Daub et al. Unpublished data 2003] and *R. coronifer *[35] of which the latter two were retrieved from NCBI (National Center for Biotechnology Information) dbEST and the NCBI Trace Archive. The source of all tardigrade samples consisted of whole adult animals except for the *H. dujardini *sample where adults and juveniles had been pooled.

**Table 1:** Summary of the expressed sequence tag (EST) analysis of the *M. tardigradum *stages (active and inactive).

Description	Active	Inactive
Total number of raw sequences	4992	4987
Total number of quality ESTs	3617	3498
Number of contigs	466	431
Number of ESTs in contigs	2103	2106
Average clone per contig	4.5	4.8
Number of singletons	1540	1437
Total non-redundant sequences	1997	1858
Blast hits (%)	52.83	51.18
No blast hits (%)	47.17	48.82

**Table 2:** Summary of number of EST sequences, contigs, and singletons in tree tardigrade cDNA libraries.

	*M. tardigradum*	*H. dujardini*	*R. coronifer*
# of raw sequences	9984	5235	3360
# of quality ESTs	7209	5221	2819
Singleton	2419	1640	1083
Contigs	864	707	373
unigene	**3283**	**2347**	**1456**

### Analysis of the *M. tardigradum *cDNA library

**Figure 1:**
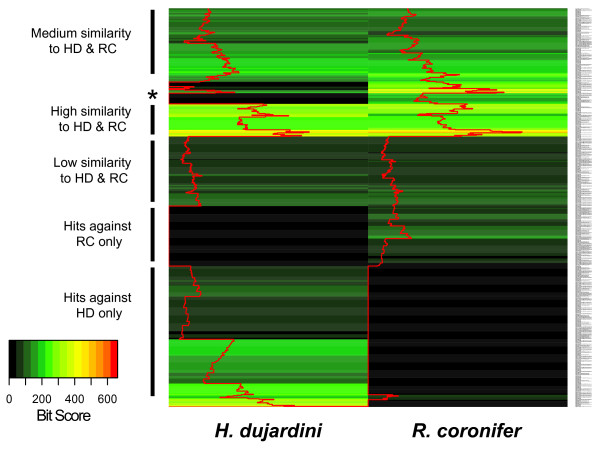
**Clustered heatmap of *M. tardigradum *TBLASTX relative bit score similarities against *H. dujardini *&* R. coronifer***. Top BLAST bit scores are shown as color gradient corresponding to their value. The red trace was added for better visualization of similarity values. The asterisk (*) marks sequences that could not be found in *H. dujardini *but are highly conserved between *M. tardigradum *and *R. coronifer*.

As summarized in Table 1, a total of 9979 clones were sequenced from the *M. tardigradum *library generated from two stages, active and inactive, in order to obtain various transcripts and to extract putative anhydrobiotic candidate genes. Assembly of the ESTs allowed the identification of 1997 and 1858 non-redundant sequences for active and inactive stages, respectively. The average unigene length was 579 nucleotides. Homology search (BLASTX) using *M. tardigradum *unigenes against the NCBI database showed that nearly 50% of the ESTs had no corresponding entry in GenBank. All ESTs were deposited in GenBank (See accession numbers in the additional file 1). The three available tardigade datasets were processed and compared (Table 2, Figure 1) in order to get an overview of the similarity and redundancy between our library and the other two EST resources.

### GO enrichment analysis of *M. tardigradum *ESTs

**Figure 2:**
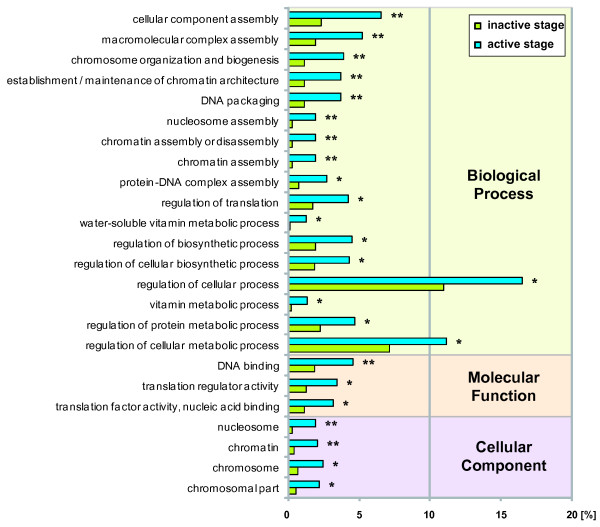
**Differentially represented Gene Ontology terms between active and inactive EST libraries of *M. tardigradum***. GO enrichment analysis between active and inactive EST libraries using Fisher's exact test with a false discovery rate (FDR) cutoff of p ≤ 0.05 (*) and p ≤ 0.01 (**). The number of transcripts associated with a specific GO term are represented as percentage of all functionally annotated EST in their respective libraries (active: N = 1207 and inactive: N = 1055).

To study the functional differences between active and inactive stages of *M. tardigradum*, we performed a GO enrichment analysis between the two datasets (Figure 2; additional file 2). Studying functional differences give insight into global mechanisms that are at work in the desiccating animals. Comparing the datasets revealed that 24 GO terms were significantly underrepresented in the inactive stage. The underrepresented GO-terms which were mapped to "nucleosome", "nucleosome assembly", "chromatin assembly or disassembly" and "chromatin assembly" (GO:0000786, GO:0006334, GO:0006333, GO:0031497) consist exclusively of transcripts coding for histones. The cellular component (CC) subset of differential terms is also solely associated with structural components of the genome, such as "nucleosome" (GO:0000786), "chromatin" (GO:0005694), "chromosome" (GO:0000785), and "chromosomal part" (GO:0044427). Finding only underrepresented terms is consistent with the global metabolic arrest of animals undergoing cryptobiosis. Histone mRNA expression is tightly linked to DNA replication and regulated by the cell cycle [39]. A study in *Caenorhabditis elegans *under anoxia showed similar adaptations such as cell cycle arrest, dephosphorylation of the histone H3 and morphological changes in the chromatin distribution [40]. A metabolic suppression could limit cellular and genomic damage by reducing the energy turnover to a minimum making the organism less susceptible to stress and therefore ensuring cell survival e.g. by decreased production of free radicals. Also GO-terms involved in translation regulation seem to be affected e.g. "regulation of translation" (GO:0006417), "translation regulator activity" (GO:0045182) and "translation factor activity, nucleic acid binding" (GO:0008135), implying modulation of translational activity as a response to desiccation.

### The most abundant ESTs in active and inactive libraries of *M. tardigradum*

The total EST count obtained by comparing the active against the inactive dataset of *M. tardigradum *is summarized in the Table 3. The relative abundance of some transcripts in the inactive stage may indicate that they have been transcribed during the desiccation process or have been stored to be translated on rehydration. Biologically, high survival rates in *M. tardigradum *are accomplished only when drying slowly at high relative humidity [16,41], suggesting that anhydrobiotic tardigrades like rotifers [42], need time to activate certain mechanisms for optimal anhydrobiosis. Probably this is because the transcription of RNAs coding for protection components has to take place. Among the genes that have a higher relative representation in the inactive stage are as follows:

**Table 3:** The most abundantly represented transcripts in the *M. tardigradum *active and inactive libraries.

Gene family	EST count		E-value
	Active	Inactive	
unknown	65	113	
cytochrome b	43	37	1E-080
intracellular fatty acid binding protein	30	43	7E-015
kazal-type serine proteinase inhibitor	24	38	7E-06
unknown	38	32	
ATP synthase F0 subunit 6	28	28	7E-017
unknown	30	21	
unknown	21	28	
unknown	19	23	
cytochrome c oxidase subunit III	21	20	4E-036
cytochrome c oxidase subunit I	8	29	5E-144
40S ribosomal protein S27	13	15	2E-038
40S ribosomal protein S25	19	13	4E-023
40S ribosomal protein S21	10	20	7E-024
NADH dehydrogenase subunit 4	13	15	2E-021
unknown	16	10	
vitellogenin	9	16	2E-013
similar to Actin-5C isoform 2	3	16	1E-015
cytochrome oxidase subunit II	8	10	2E-034
unknown	5	12	
cystatin B	5	10	5E-014
NADH dehydrogenase subunit 5	4	10	3E-021
elongation factor 1 alpha	10	2	4E-124

### Lipid-related transcripts

Lipid-related transcripts are represented mainly by intracellular fatty acid binding protein (FABP). FABPs have a low molecular mass and bind with high affinity to hydrophobic ligands such as saturated and unsaturated long-chain fatty acids. Various functions have been proposed for FABPs such as the uptake, transport, and delivery of fatty acids to beta-oxidation [43,44]. FABPs are also thought to be active fatty acid chaperones by protecting and shuttling fatty acids within the cell [45,46]. However the biological role and mechanisms of action of FABPs remain poorly understood. The transcript level of FABP was identified by cDNA array and Northern blot analysis as being up-regulated during hibernation of ground squirrels [43,47]. Members of the FABPs family have recently been identified and reported to increase in the monogonont rotifer *Brachionus plicatilis *during dormancy [48]. The presence of FAPB in inactive stage of *M. tardigradum *may imply conserved mechanisms shared between rotifer dormancy and anhydrobiosis in tardigrades and presumably other organisms as well. FAPB may protect membranes and ensure fatty acids as energy saving storage during anhydrobiosis.

### Protease inhibitors

To date, little is known about the possible mechanisms of proteolytic inhibition or suppression in anhydrobiotic organisms. Protease inhibitors are candidate genes which would offer protection against protein degradation during anhydrobiosis. Among the abundant protease inhibitors transcripts in inactive stages of *M. tardigradum *are Kazal-type serine proteinase inhibitor and Cystatin B. Overexpression of Cystatin B (an intracellular cysteine proteinase inhibitor) in transgenic yeast and *Arabidopsis *showed an increase in the resistance to high salt, drought, oxidative, and cold stresses [49]. Elevated levels of transcripts coding for protease inhibitors such as Cystatin B have also been found in brine shrimp cysts [50]. The abundance of protease inhibitors may inhibit proteolytic reactions of proteases that could damage tissues during the desiccation process or as a response to induction of proteases as a result of aggregated proteins. Also a protection against microbial degradation could be possible as this can occur at humidity levels at which tardigrades can't rehydrate and actively mobilize any cellular defence mechanisms.

### Cytochrome c oxidase subunit I

Cytochrome c oxidase subunit I (COXI) is a mitochondrial gene that encodes the cytochrome c oxidase subunit I, a crucial enzyme involved in oxidative phosphorylation and thus energy production. COXI was over threefold more represented in the inactive state. Transcripts encoding COXI were also abundantly expressed during dehydration stress in the antarctic nematode *Plectus murrayi *[51] and up regulated by temperature increase in the yeast-like fungus *Cryptococcus neoformans *[52]. The mitochondrial COXI upregulation may serve to prevent the damage to the electron transport chain caused by desiccation and to keep an increased energy production for the survival of the tardigrades.

The sequences, which could not be assigned any function based on homology search in NCBI, were searched for conserved domains in ProDom [53] and Swiss-Prot databases [54] but did not show any hits. Since these are not all full-length sequences, it is possible that they may have missed characteristic motifs or domains for classification. A detailed investigation of their function as well as other identified transcripts presented in Table 3 will be a task in the future.

### Transcripts with putative functions in desiccation resistance identified in all three tardigrade species datasets

In a cross-search over the four tardigrade EST resources (active and inactive libraries of *M. tardigradum*, *R. coronifer *and *H. dujardini*), transcripts which are potentially associated with desiccation tolerance during anhydrobiosis in other organisms were identified (see additional files 3, 4 and 5).

### Detoxification-related genes

Oxidative stress proteins have been shown to be an important component in many biological processes [55]. They mediate detoxification and have putative roles as antioxidants such as glutathione S-transferase (GST), thioredoxin, superoxide dismutase (SOD), glutathione peroxidases and peroxiredoxin. It was shown that overexpression of GST/glutathione peroxidase increased the resistance to oxidative and water stress in transgenic tobacco plants [56]. GSTs are a diverse superfamily of multifunctional proteins that are reported to play a prominent role in the detoxification metabolism in nematodes [57]. In particular the up-regulation of detoxifying enzymes GST and SOD in *Plectus murrayi *[51] suggests an efficient role of reactive oxygen species (ROS) scavenging mechanisms under desiccation stress. These observations led us to postulate that the tardigrade GST and SOD are likely to deal with oxidatively damaged cellular components during desiccation. These enzymes that help in the removal of these compounds contribute to cellular survival after oxidative damage.

### Aquaporins

Many organisms adapt to desiccation stress by the activation of various water-channel proteins, called aquaporins (AQP) [58,59]. Data from *Polypedilum vanderplanki *indicates that of the two aquaporins isolated from this organism, one is involved in anhydrobiosis, whereas the other controls water homeostasis of the fat body during normal conditions [60]. Similarly, the aquaporins in larvae of the goldenrod gall fly, *Eurosta solidaginis *were either upregulated (AQP3) or downregulated (AQP2 and AQP4) following desiccation [61]. The upregulated AQP3 is especially intriguing because it is permeable to water and glycerol across the cell membrane as larvae prepare for the osmotic stress associated with desiccation. In our study aquaporin transcripts have been identified in all tardigrade datasets. These AQPs may act in concert with other transmembrane proteins to mediate the rapid transport of water across the plasma membrane during anhydrobiosis when its diffusion through the phospholipid layer of the membrane is limited.

### Molecular chaperones

In the four tardigrade datasets we have identified some putative heat shock protein (HSP) encoding genes. HSPs are highly conserved throughout evolution and they function as molecular chaperones and play primary roles in protein biosynthesis and folding [62]. In tardigrades, there is considerable debate concerning the role of HSPs under desiccation stress. In the *R. coronifer*, a lower level of Hsp70 protein was found in desiccated animals when compared with active ones [63]. In *M. tardigradum*, one isoform of the *hsp70 *tarnscripts showed up-regulation during the transition from active to the inactive state [64,65], while the other *hsp70 *isoforms are downregulated and seem not to be directly involved in anhydrobiosis. Using the same model *M. tardigradum*, Reuner et al. [65] found an upregualtion of hsp90 in the inactive state. Certainly Hsp70 isoforms and hsp90 are involved in tardigrade desiccation, but further studies are necessary to understand how these proteins work to protect anhydrobiotic organisms.

Much attention was recently paid on the chaperone-like LEA (late embryogenesis abundant) proteins in anhydrobiotic animals [66,67]. LEA proteins are mainly low molecular weight (10-30 kDa) proteins associated with tolerance to water stress resulting from desiccation and cold shock [68,69]. Genes encoding LEA-like proteins have been identified in the nematode *Aphelenchus avenae *under dehydration condition [70-72]. A similar gene was identified and upregulated in the larvae of *P. vanderplanki *by water stress imposed by either desiccation or hypersalinity [73]. Recently, LEA have also been identified and shown to be induced under dehydration in the springtail *Megaphorura arctica *[74]. In the tardigrade EST libraries, LEA transcripts have been found in the *H. dujardini *library (the less tolerant tardigrade) and also in the proteome map of *M. tardigardum *[75]. These data suggest that LEA-like proteins could be widespread in anhydrobiotic organisms and serve important functions during desiccation.

The translationally controlled tumor protein (TCTP) found in all tardigrada datasets is often designated as a stress-related protein because of its highly regulated expression in stress conditions and its close relation to a family of small chaperone proteins [76]. Importantly, TCTP can bind to native proteins and protect them from thermal denaturation [77].

### Trehalose synthesis-related gene

Trehalose, which accumulates in many anhydrobiotic organisms during desiccation is proposed to act as a common water replacement molecules and stabilizer of biological structures [78-80]. The accumulation of trehalose has been reported in the cysts of the crustacean *Artemia franciscana *[81], in the nematode *Aphelenchus avenae *[82] and in the insect larvae of the *P. vanderplanki *[83]. However, anhydrobiotic Bdelloid rotifers are unable to produce trehalose [41,84]. In addition, the trehalose-6-phosphate synthase genes (*tps*) have not been found in rotifer genomes [41]. Although trehalose accumulates substantially in the eutardigrade *Adorybiotus coronifer *[85], it was surprisingly immeasurable in *M. tardigardum *[16] and we could not find transcripts of *tps *in *M. tardigradum *ESTs. Nevertheless, transcripts coding for trehalases have been described in *M. tardigradum *[86] but we propose that its function is probably limited to the catabolism of trehalose taken up from food sources. The hypothesis of trehalose as a protective agent during desiccation may not be applicable to all anhydrobiotic organisms and in *M. tardigradum *other strategies are probably employed.

### Comparative ESTs analysis between the three tardigrade species

The datasets analysed in this study represent most of the available transcriptome data from tardigrades, and until now there is little information on tardigrade genome and transcriptome structure. The genome sizes range from very compact genomes, ~75 Mb for *H. dujardini*, considered as one of the smallest tardigrade genomes [36], up to 800 Mb for other species http://www.genomesize.com. Our dataset adds a substantial part towards the complete gene content in the tardigrada species.

To investigate the complementation of the three tardigrade datasets (*M. tardigradum*, *H. dujardini *and *R. coronifer*) we searched for putative orthologous sequences across all three datasets. Using a TBLASTX search with an e-value threshold of 10^-5 ^we compared the *M. tardigradum *unigenes against the other two datasets. The BLAST bit-score of each top-scoring hit was extracted and *M. tardigradum *sequences that exhibited sequence similarity against at least one other tardigrade species are presented as a clustered heatmap in Figure 1 (see also additional file 6). *M. tardigradum *unigenes show similarities against both other species with some hits only present in either one of them (N = 785). A higher coverage of *M. tardigradum *transcripts can be seen in the *H. dujardini *dataset compared to *R. coronifer *which is likely due to the smaller *R. coronifer *dataset. This cross species comparison implies that the remaining 2498 unigenes contained in the *M. tardigradum *dataset represent further yet unknown tardigrade transcripts and expands the known tardigrade sequence data. These might be very interesting for studying the evolutionary relationships of protein families.

To calculate the average relative transcriptome sequence similarity between *M. tardigradum *and the other two tardigrade species we included only sequences that were common to all three tardigrade species (N = 368). These contained mainly abundant transcripts e.g. ribosomal proteins, ADP-ribosylation factor, ubiquitin, glyceraldehyde-3-phosphate dehydrogenase and heat shock proteins. The resulting average transcript similarity for *M. tardigradum *against *H. dujardini *was 147.66 +/- 88.27 and *M. tardigradum *against *R. coronifer *150.95 +/- 93.74. This is reflected in the phylogenetic distance calculated using 18S rRNA sequences (see additional file 7), which positions *R. coronifer *closer to *M. tardigradum*.

## Conclusions

This study describes novel sequence data from the tardigrade *M. tardigradum *that identified a set of 3283 unigenes, which significantly contributes to the available tardigrade sequence data and will help to establish this tardigrade as a model for studying desiccation tolerance. The comparison of active and inactive stage EST libraries by performing an exploratory GO enrichment analysis suggests a metabolic suppression in terms of replication and translation during desiccation. The tardigrade-EST resource generated from this study will serve as a reference for future global gene expression experiments, aiming at the identification of key regulators of desiccation resistance during anhydrobosis. Furthermore the datasets of *H. dujardini *and *R. coronifer *will serve as additional resources that could give clues about the evolutionary conservation of these regulators between tardigrade species of different anhydrobiotic capabilities.

## Methods

### Animal culture and sampling

*M. tardigradum *was reared in a laboratory culture on 3% agar plates covered with Volvic^® ^mineral water at 20 ± 2°C and a light/dark cycle of 12 h as previously published [8]. For all experiments, adult animals (eight weeks after hatching) in good condition were collected directly from the agar plate using a pipette and a stereomicroscope. Tardigrades were starved for 3 days, and washed for several times with Volvic^® ^mineral water before being processed to avoid contaminations. A total of 1000 animals were collected into 1.5 ml Eppendorf tubes in aliquots of 200 animals each. Animals representing the active state were frozen directly in liquid nitrogen. Anhydrobiotic stages of *M. tardigradum *were generated by a previously published protocol [8]. Briefly, *M. tardigradum *(200 animals) were placed in 1.5 ml Eppendorf tube and desiccated at room-temperature at 85% relative humidity (RH) for 12 to 16 hours (till they have completed the tun formation) and then at 35% RH for further 48 hours. The animals were frozen at -80°C until their experimental use.

### Library construction

Total RNA extraction was performed by following the instructions of QIAGEN RNeasy R Mini kit (Qiagen, Hilden, Germany). Complete lysis of the tardigrades and especially disruption of their harsh cuticle was achieved by sonication on ice for 1 min (duty cycle 0.5s) by using a microsonicator (Probe 73, Sonopuls; Bandelin). For cDNA synthesis 1 μg total RNA was reverse transcribed using the Creator™ SMART™ cDNA Library Construction Kit following the manufacturers recommendations (Clontech-TaKaRa Bio Europe, France). The resulting first strand cDNA was amplified by LD-PCR for 18 cycles according to the manufacturers protocol using the 5' PCR primer (5'-AAGCAGTGGTATCAACGCAGAGT-3') as the forward and the CDSIII/3'PCR Primer (5'-ATTCTAGAGGCCGAGGCGGCCGACATG-d(T)30N-1N-3') as reverse primer. The amplified PCR products were then analyzed by agarose gel electrophoresis. After digestion of the amplified cDNA with the *Sfi*I restriction enzyme, products smaller than 300 bp were removed using the Chroma Spin-400 column as described in the Creator SMART™ protocol and cloned into pDNR-Lib cloning vector. This procedure was chosen because of the low amount of starting material. Plasmids were transferred via electroporation to *Escherichia coli *(strain DH10B, Invitrogen, Karlsruhe, Germany).

### cDNA sequencing

In total, 9984 cDNA clones were either picked by hand or automatically using a QPix robot (Genetix, UK) into 384 well LB-agar culture plates containing chloramphenicol. Sequencing was mostly from the 5' end using standard M13 forward sequencing primer. The sequencing of the cDNA library was sequenced on a ABI 3730XL capillary sequencer by GATC Biotech AG (Konstanz, Germany).

### Sequence analysis and annotation

**Figure 3:**
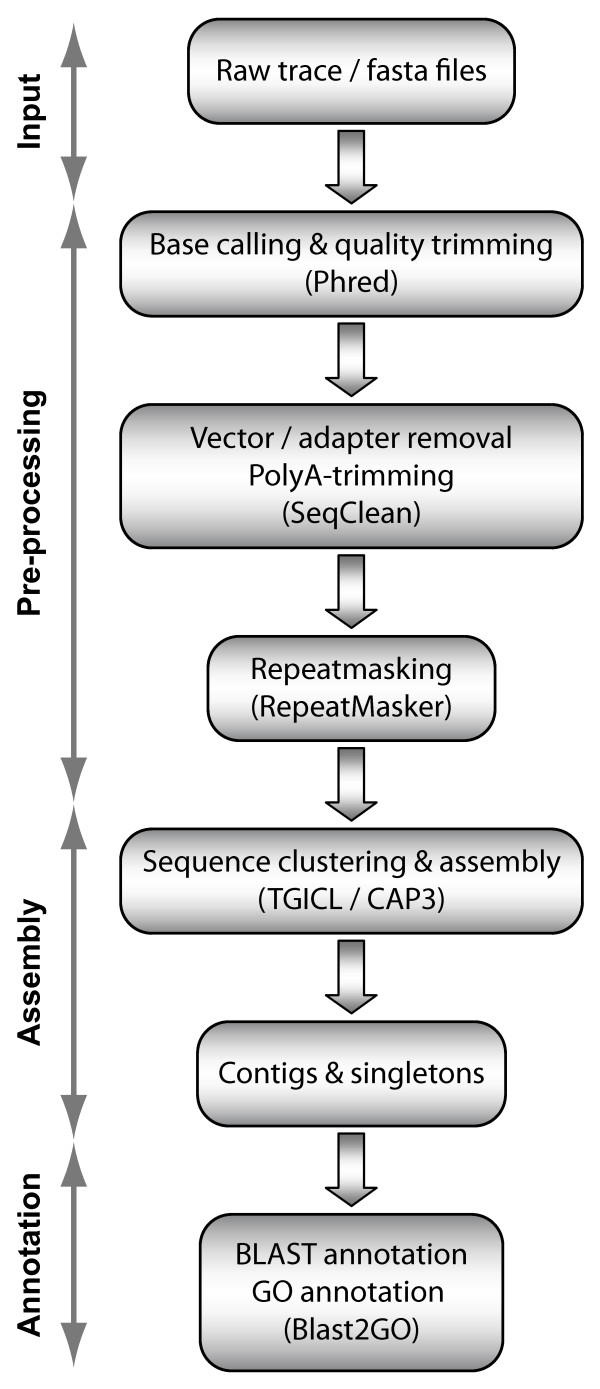
**Overview of the tardigrade EST processing pipeline**. Software used by each process in this flowchart are shown in brackets.

The EST analysis pipeline (Figure 3) includes typically, EST pre-processing, EST assembly and annotation of the resulting unigenes. The result is the generation of a clean, high-quality EST sequence set. Both chromatogram (*M. tardigradum*) and FASTA sequences (*H. dujardini *and *R. coronifer*) files are accepted as entry point to the analysis. Base calling was performed with phred [87,88] using a score threshold of 20. Low quality sequences, cloning vector, poly A or T tails, adaptors, and short sequences (<100 base pairs) are removed from the sequences with SeqClean [89]. Repetitive elements are masked with RepeatMasker [90]. Sequences that can be considered contaminants and unexpected vector sequences are also removed with SeqClean, using NCBI's UniVec database (v5.1) [91].

TIGR Gene Indices clustering tools (TGICL) with standard parameters [92] and CAP3 [93] have been used for the assembly step. For functional annotation, processed putative unique transcripts were loaded into the Blast2GO software [94]. Blasting was done with BLASTX algorithm using Blast2GO (v2.3.5) standard parameters. Unigenes were annotated with GO terms using standard evidence GO weight parameters. The 'Augment Annotation by ANNEX' function was used to refine annotations. Subsequently, Inter-ProScan [95] was performed to find conserved functional domains. GO terms derived from domains were merged into the existing GO annotation of the respective unigenes.

### GO enrichment analysis

Identification of GO terms differentially enriched between the active and inactive *M. tardigradum *datasets was performed using the GOSSIP statistical framework [96] webservice via the BLAST2GO software. GOSSIP employs 2 × 2 contingency tables of annotation frequencies for each GO term and computes p-values using Fisher's exact test. The statistical framework accounts for false positives (type-I-errors) that arise from multiple testing by calculating adjusted p-values. We screened for significantly enriched GO-terms by controlling the false discovery rate (FDR), setting a cut-off threshold of pFDR(p) ≤ 0.05. GO terms fulfilling this criterion were considered differentially enriched between the two *M. tardigradum *datasets.

## Authors' contributions

BM established and optimized the tardigrade RNA extraction protocol and constructed and managed the cDNA clone libraries, MG performed functional annotation and enrichment analysis, putative orthologue prediction and gave useful comments on sequence analysis, MF was responsible for oversight, budget, obtaining the funding for the project, and contributing advice at each step of the research. FF performed quality control, processing and assembly of ESTs and was involved in data analysis, TD contributed to the bioinformatic analysis. WW performed the phylogenetic analysis, RS provided the animals and coordinated the project and contributed comments on candidate anhydrobiotic genes, MS and DR supported the identification of anhydrobiotic genes. BM and MG wrote the main part of the manuscript. All authors read and approved the final manuscript.

## Supplementary Material

Additional file 1 **List of *M. tardigradum *ESTs and their GenBank accession numbers**. This file provides a list of dbEST ID, User ID and GenBank accession numbers of all *M. tardigradum *ESTs. Click here for file

Additional file 2 **GO-enrichment analysis statistics of *M. tardigradum *datasets**. This file contains details about the GO-enrichment analysis between the active and inactive stage of *M. tardigradum *using GOSSIP. A list of enriched ESTs is provided. Click here for file

Additional file 3 **Putative anhydrobiotic transcripts identified in *M. tardigradum *dataset**. This file provides a list of ESTs in active and inactive stages of *M. tardigradum *that are potentially associated with desiccation tolerance. Click here for file

Additional file 4 **Putative anhydrobiotic transcripts identified in *H. dujardini *dataset**. This file provides a list of *H. dujardini *sequences that are potentially associated with desiccation tolerance. Click here for file

Additional file 5 **Putative anhydrobiotic transcripts identified in *R. coronifer *dataset**. This file provides a list of *R. coronifer *sequences that are potentially associated with desiccation tolerance. Click here for file

Additional file 6 **Putative orthologous sequences of *M. tardigradum *against *H. dujardini *and *R. coronifer***. This file provides a list of the putative orthologues shared by tardigrade species investigated in this study. Click here for file

Additional file 7 **Phylogenetic tree based on tardigrade 18S rRNA sequences**. Displays a phylogenetic tree constructed from *E. testudo*, *M. tardigradum*, *R. coronifer *and *H. dujardini *18S rRNA sequences. Click here for file
