# Supplementary material for: Transcriptome survey of the anhydrobiotic tardigrade Milnesium tardigradum in comparison with Hypsibius dujardini and Richtersius coronifer
Source: BMC Genomics. 2010 Mar 12;11:168. doi: 10.1186/1471-2164-11-168 (PMC2848246; doi:10.1186/1471-2164-11-168)
Supplement: Additional file 7 — Phylogenetic tree based on tardigrade 18S rRNA sequences. Displays a phylogenetic tree constructed from E. testudo, M. tardigradum, R. coronifer and H. dujardini 18S rRNA sequences. [file 1471-2164-11-168-S7.PDF]

Additional file 6: Phylogenetic analysis based on 18S rRNA sequences

The multiple sequence alignment was prepared with ClustalW2 [1] from 18S rRNA sequences retrieved from GenBank. Accession numbers: *E. testudo* (DQ839607.1), *M. tardigradum* (U49909.1), *R. coronifer* (DQ839604.1), *Hypsibius sp.* (EU266939.1). Data mining in the *H. dujardini* dataset identified a 508 bp fragment that was 100% identical to EU266939.1 (1671 bp). For constructing a more robust phylogenetic tree the longer sequence of EU266939.1 was used.
Phylogenetic and molecular evolutionary analyses were conducted using *MEGA* version 4 [2]. Confidence levels were evaluated by bootstrap analysis with 1000 replicates. The phylogenetic tree was calculated using Neighbour Joining method and was rooted using the heterotardigrade *E. testudo* as an outgroup (figure 1). The distance values are summarized in table 1. The calculated genetic distance shows that the sequence divergence is smaller between *M. tardigradum* and *R. coronifer* (9%) than *M. tardigradum* and *H. dujardini* (11.3%).

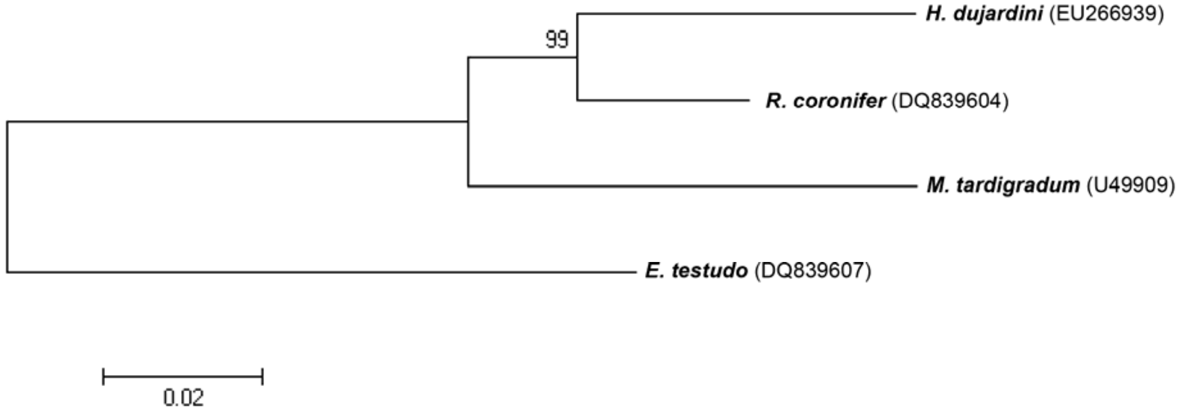

Figure 1 - Phylogenetic tree of 18S rRNA sequences from four tardigrade species by Neighbour Joining method. Bootstrap value is given as number above fork. GenBank accession numbers in brackets.

Table 1 - The distance matrix of studied 18S rDNA sequences. Distances were calculated using MEGA v4.

	M. tardigradum	*R. coronifer*	*H. dujardini*	*E. testudo*
M. tardigradum		0.090	0.113	0.190
R. coronifer	0.090		0.064	0.170
H. dujardini	0.113	0.064		0.189
E. testudo	0.190	0.170	0.189	

References

1. Larkin MA, Blackshields G, Brown NP, Chenna R, McGettigan PA, McWilliam H, Valentin F, Wallace IM, Wilm A, Lopez R, Thompson JD, Gibson TJ, Higgins DG: **Clustal W and Clustal X version 2.0**. *Bioinformatics* 2007, **23:**2947-2948.
2. Tamura K, Dudley J, Nei M, Kumar S: **MEGA4: Molecular Evolutionary Genetics Analysis (MEGA) Software Version 4.0** *Mol Biol Evol* 2007, **24**:1596–1599.
